# New fungal primers reveal the diversity of Mucoromycotinian arbuscular mycorrhizal fungi and their response to nitrogen application

**DOI:** 10.1186/s40793-024-00617-x

**Published:** 2024-09-18

**Authors:** Mirjam Seeliger, Sally Hilton, George Muscatt, Christopher Walker, David Bass, Felipe Albornoz, Rachel J. Standish, Neil D. Gray, Louis Mercy, Leonidas Rempelos, Carolin Schneider, Megan H. Ryan, Paul E. Bilsborrow, Gary D. Bending

**Affiliations:** 1 School of Natural and Environmental Sciences, Newcastle University, Newcastle upon Tyne, NE1 7RU UK; 2 School of Life Sciences, University of Warwick, Coventry, CV4 7AL UK; 3 Royal Botanic Gardens Edinburgh, 21A Inverleith Row, Edinburgh, EH3 5LR UK; 4 UWA School of Agriculture and Environment, University of Western Australia, Crawley, WA 6009 Australia; 5 Centre for Environment, Fisheries, and Aquaculture Science, Barrack Road, The Nothe, Weymouth, DT4 8UB UK; 6 Department of Life Sciences, The Natural History Museum, Cromwell Road, London, SW7 5BD UK; 7 Commonwealth Scientific and Industrial Research Organisation, Land and Water, Wembley, WA Australia; 8 School of Environmental and Conservation Sciences, Murdoch University, South Street, Murdoch, WA 6150 Australia; 9 INOQ Gmbh, 29465 Schnega, Germany; 10 Present Address: Micropathology Ltd, Coventry, CV4 7EZ UK

**Keywords:** Arbuscular mycorrhizas, Fine root endophytes, Mucoromycotina, Nitrogen

## Abstract

**Background:**

Arbuscular mycorrhizas (AM) are the most widespread terrestrial symbiosis and are both a key determinant of plant health and a major contributor to ecosystem processes through their role in biogeochemical cycling. Until recently, it was assumed that the fungi which form AM comprise the subphylum Glomeromycotina (G-AMF), and our understanding of the diversity and ecosystem roles of AM is based almost exclusively on this group. However recent evidence shows that fungi which form the distinctive 'fine root endophyte’ (FRE) AM morphotype are members of the subphylum Mucoromycotina (M-AMF), so that AM symbioses are actually formed by two distinct groups of fungi.

**Results:**

We investigated the influence of nitrogen (N) addition and wheat variety on the assembly of AM communities under field conditions. Visual assessment of roots showed co-occurrence of G-AMF and M-AMF, providing an opportunity to compare the responses of these two groups. Existing ‘AM’ 18S rRNA primers which co-amplify G-AMF and M-AMF were modified to reduce bias against Mucoromycotina, and compared against a new ‘FRE’ primer set which selectively amplifies Mucoromycotina. Using the AM-primers, no significant effect of either N-addition or wheat variety on G-AMF or M-AMF diversity or community composition was detected. In contrast, using the FRE-primers, N-addition was shown to reduce M-AMF diversity and altered community composition. The ASV which responded to N-addition were closely related, demonstrating a clear phylogenetic signal which was identified only by the new FRE-primers. The most abundant Mucoromycotina sequences we detected belonged to the same Endogonales clades as dominant sequences associated with FRE morphology in Australia, indicating that closely related M-AMF may be globally distributed.

**Conclusions:**

The results demonstrate the need to consider both G-AMF and M-AMF when investigating AM communities, and highlight the importance of primer choice when investigating AMF community dynamics.

**Supplementary Information:**

The online version contains supplementary material available at 10.1186/s40793-024-00617-x.

## Background

Arbuscular mycorrhizal fungi (AMF) are considered to form the most common and important symbiosis in terrestrial ecosystems [65]. Fossilised spores and hyphae in plant roots from the Ordovician date the first association of fungi with land plants to 460 million years ago and provide evidence for the significance of mycorrhizas in the evolution of vascular plants [46, 57]. Arbuscular mycorrhizas (AM) are traditionally considered to be formed by fungi within the monophyletic Glomeromycotina subphylum [59]. However, more recently a further group of arbuscle-forming fungi has been recognised within the Mucoromycotina subphylum [41, 69]. The species complex of the distinctive ‘fine root endophyte’ (FRE) arbuscular fungi was formerly collected within the species *Glomus tenue* within Glomeromycotina [17, 62], but molecular analyses have shown that these fungi are phylogenetically different to Glomeromycotina AMF (G-AMF; [41]). Consequently, *Glomus tenue* (basionym *Rhizophagus tenuis*) was renamed *Planticonsortium tenue* [69] and the new genus resides within the Endogonales order within the subphylum Mucoromycotina [2, 3, 40]. To date this is the only described species of M-AMF. Co-occurrence of both G-AMF and Mucoromycotina AMF (M-AMF) has frequently been reported across plant species and environments [41, 52]. The detection of both M-AMF and G-AMF in fossils of early land plants from the Rhynie Chert (ca. 407 million years ago) indicates early co-occurrence of these two groups in plant-fungal associations [60].

Several factors have resulted in the occurrence of M-AMF in nature, and their contributions to ecosystem processes, being overlooked. DNA primers designed to profile AMF communities have been targeted at G-AMF and fail to amplify M-AMF [41]. Furthermore, spores of M-AMF are much smaller (≤ 20 µm) than those of G-AMF (30 µm–1 mm), and as a result M-AMF are not recorded during fungal community assessments using spore morphology [41]. The characteristic fine hyphae (0.1–4 µm) and fan-shaped arbuscules enable differentiation of M-AMF from G-AMF by microscopy [33, 40, 41]. But this requires high power microscopy, such that M-AMF are not detected in studies which assess root colonisation under low magnification. M-AMF have thinner cell walls than G-AMF and form hyphal ropes, but the two groups of fungi have similar colonisation behaviour and ultrastructural features [2, 14].

Analysis of the distribution of FRE indicates that like G-AMF, M-AMF are globally distributed and found in both natural and agricultural soils, although there are indications that M-AMF are absent from tropical and subtropical biomes [4, 40]. Regardless, their functional significance is far less understood than that of G-AMF [40]. Recently, nutritional mutualism involving exchange of fungal phosphorus (P) and nitrogen (N) in return for plant carbon (C) has been shown for M-AMF in axenic systems [20]. Furthermore, comparative studies with G-AMF in association with liverworts showed higher N-uptake by M-AMF, while the reverse was true for P, suggesting that these groups of fungal endophytes could have complementary roles in nutrient acquisition [13].

Agricultural practices such as N-addition have negative effects on biodiversity in plant [9] and soil microbial [26, 74] communities and cause wider environmental impacts such as eutrophication of surface and groundwater bodies [10]. In particular, N-addition can decrease abundance of G-AMF in soil and roots of agricultural plants [15] and result in proliferation of ‘weedy’ G-AMF in natural systems [5]. In agricultural soils, optimised N-management is important to promote G-AMF diversity [30], and can be decisive for the success of G-AMF inoculation [12].

In contrast to the wealth of knowledge on the impacts of agricultural management practices and environmental variables on the diversity and community composition of G-AMF [15, 48], understanding of the responses of M-AMF are largely limited to morphological root colonisation data with a dominance of data from Australia and New Zealand [41]. Recently, Albornoz et al. [3] showed that M-AMF and G-AMF communities had similar responses to key environmental variables, including soil P concentration and pH within regionally co-located pasture sites. However, a continent-wide analysis across multiple ecosystems provided clear evidence for different responses of the two groups of fungi to edaphic and climatic variables, indicating that these groups of fungi have distinct but overlapping ecological niches [4]. In wheat and field pea in southern Australia, Ryan and Kirkegaard [51] showed that P-addition decreased the colonisation of M-AMF in roots more so than that of G-AMF, while, in the glasshouse Jeffery et al. [21] showed that the impact of 5 rates of P addition on colonisation of pasture legumes differed between M-AMF and G-AMF. However, the role of soil P, let alone soil N, in shaping M-AMF communities has received little attention. Sigüenza et al. [56] indicated that M-AMF root colonisation may be favoured by high N environments, and furthermore at the continental scale, relative abundance of M-AMF was favoured by availability of soil mineral-N [4]. Given the potentially contrasting contributions of G-AMF and M-AMF to host N nutrition [13], there is a need for comparative understanding of the role of N in shaping the diversity and composition of these contrasting AM communities.

The importance of host variety for determining G-AMF colonisation and growth responses has been demonstrated in many crops such as clover [53], onion [61], potato [1], sorghum [29], maize [55] and durum wheat [12]. However, most evidence for variety selection of G-AMF comes from studies of common wheat (*Triticum aestivum*. Host variety-dependent variations in mycorrhizal colonisation of wheat have been widely reported (Azcón and Ocampo 1981) [19, 27] and have been linked to nutrient use efficiency [24, 63] and abiotic stress tolerance [28]. There has been a long-term debate about the breeding background which determines mycorrhizal responsiveness of wheat [19, 27, 35, 75], and the results are inconclusive. However, most plant variety-differences in AMF selection are based on root colonisation assessments, and less is known about impacts on mycorrhizal community composition [61]. Mao et al. [34] provided evidence that G-AMF community assembly dynamics in wheat can operate at a variety level, with 21 varieties showing significantly different G-AMF mycobiomes under field conditions, and that varietal composition was correlated with drought stress tolerance. However, these variations had no effects on wheat performance [34]

There are several studies which report morphological evidence for M-AMF symbioses in agricultural crops, including wheat [18, 51, 52, 58], but to our knowledge, relationships between M-AMF and plant variety has not been investigated. Most recent studies on M-AMF in agricultural systems come from southern Australia where wheat was shown to have an AM fungal community dominated by M-AMF (present in 80% of AM colonised root length) abundance of which was impacted by previous crop and P-addition [51]. However, at the community level, understanding of M-AMF in agricultural systems is limited, and is contributed primarily from a survey of subterranean clover in pasture systems across southern Australia [3], and additional data from single fields of a broad range of crops (from wheat to fruit orchards) across a range of biomes in Albornoz et al. [4]. In all cases root associated communities contained multiple putative M-AMF taxa, but the taxa richness was lower than G-AMF communities [3, 4, 40]. Interestingly, the survey across Australian biomes by Albornoz et al. [4] found putative M-AMF had greater distribution and abundance in the soils of agricultural fields than adjacent natural ecosystems, indicating that, in contrast to G-AMF, agricultural practices may favour M-AMF.

In this study we investigated the influence of N-addition on the assembly of AM communities in two contrasting wheat cultivars, under field conditions. Preliminary visual assessment of roots at the field site showed co-occurrence of G-AMF and M-AMF, which provided an opportunity to compare the responses of these two groups of fungi to both crop variety and N-addition. To date, work investigating M-AMF community composition has used 18S rRNA primers designed by Sato et al. [54] which co-amplify M-AMF and G-AMF, but provide poor coverage of putative M-AMF and short (220 bp) sequence lengths [3, 4, 33, 41]. In the current study we modified the Sato forward primer to reduce bias against M-AMF, and designed new 18S rRNA Mucoromycotina specific primers which amplified longer sequences (440 bp), thus providing improved resolution of community composition and phylogeny.

## Methods

### Field site and agronomic management

The field trial was conducted in 2018–2019 at Nafferton Experimental Farm in Northumberland (54° 59′ 27.26″ N, 1° 54′ 26.96″ W, Stocksfield, UK). The soil type at the experimental field site is a uniform sandy clay loam [47] with a pH of 6.8 and Olsen extractable contents of 8.1 ± 1.49 mg L^−1^ P, 72 ± 8.86 mg L^−1^ potassium and 157 ± 6.18 mg L^−1^ magnesium. The interaction of N application and wheat variety was assessed in a randomised-block split-plot factorial design. There were three 24 × 24 m blocks across a 0.3 ha area. Each block contained a 6 m × 4 m plot of each the 4 treatments (i.e. (1). Aszita with no added N (n = 3) (2). Aszita with added N (n = 3) (3). Skyfall with no added N (n = 3) and (4). Skyfall with added N (n = 3). The factorial design provided 6 plots for assessing the effect of both N treatment and variety on fungal communities, with 3 replicates for each variety/ fertiliser combination. The winter wheat varieties Skyfall (released 2014 by RAGT Seeds Ltd., UK) and Aszita (released in 2004 by Getreidezüchtung Peter Kunz, Switzerland) were drilled in September 2018.

Skyfall has a conventional breeding background, and is a modern, high yielding, semi-dwarf variety, and one of the most widely grown winter wheats in the UK. Aszita has an organic breeding background and is characterised by long straw growth of more than 1 m and achieves low grain yields, but with high quality. Both varieties have been investigated in other studies on variety-dependent AM interaction: Skyfall acquired more P through AMF in comparison to two other modern wheat varieties and showed an intermediate root length colonisation of 34% in a study by Elliott et al. [11]. Another study using the same varieties identified Skyfall as particularly dependent on AM-mediated nutrient uptake [63]. Aszita on the other hand was revealed as one of the least colonised by AMF among 94 wheat varieties in a greenhouse screening study by Lehnert et al. [28].

Half of each wheat variety population was treated with fertiliser in the form of mineral-N (ammonium nitrate; Nitram 34.5% N, CF fertilisers UK Ltd.), which was added at a rate of 170 kg N ha^−1^ in two applications: 70 kg ha^−1^ in mid-April 2019 and 100 kg 2 weeks later. The control plots did not receive any fertiliser input and are further referred to as zero-N plots. Fungicides, herbicides and plant growth regulators were applied throughout the cropping season (as detailed in Table S1).

### Root colonisation assessment

Root samples were collected from the topsoil layer of the twelve plots just after the start of stem elongation (growth stage 32, [73]) in May 2019. Shoots across a 0.25 m^2^ sampling area were cut at the stem base, prior to digging plants out. Roots from 15 to 20 cm soil depth were removed from 5–6 plants and pooled to provide a composite sample from each plot. Root samples were washed in tap water and stored in 50% ethanol until further processing. Roots were stained using the ink-vinegar method described by Vierheilig et al. [68]. First, roots were rinsed in tap water to remove ethanol residues. Then, samples were incubated in 10% KOH solution at 80 °C for two hours in the oven. Roots were rinsed with tap water and subsequently incubated in an 8% acetate/5% China ink solution overnight. Microscopy for the visual assessment of root colonisation was conducted on a total of 30 cm root length from each sample. Percentages of overall mycorrhizal frequency intensity as well as intensity of arbuscules, vesicles and hyphae were estimated based on the method described by Trouvelot et al. [66] using the INOQ Calculator Advanced [37].

### DNA extraction and sequencing

DNA was extracted from 0.5 g of the root samples using the DNeasy PowerSoilPro Kit (Qiagen, Germany) following the manufacturer’s protocol. Prior to DNA extraction, samples were homogenised using a FastPrep-24™ (MP Biomedicals, USA) at 6 m s^−1^ for 2 × 40 s periods and an incubation step for 5 min at 4 °C between homogenisations. DNA concentrations were measured by fluorometric quantification (Qubit™ Fluorometer 3.0) using the Qubit™ dsDNA high sensitivity assay kit (Invitrogen, USA) following the manufacturer’s protocol. Amplicons were produced using two different primer sets targeting the 18S rRNA gene.

The first primer set (further referred to as AM-primers) targeted both Glomeromycotina and Mucoromycotina and was adapted from the primers published by Sato et al. [54] which amplify a 220 bp region of the 18S rRNA gene (Fig. S1). Alignment of Mucoromycotina and Glomeromycotina 18S rRNA sequences showed that the Sato et al. AMV4.5NF forward primer (AAGCTCGTAGTTGAATTTCG) had 2 nucleotide differences to Mucoromycotina at the 3′ end which drastically reduced amplification of Mucoromycotina relative to Glomeromycotina (Fig. S2). The redesigned AM forward primer AM-Sal-F (AAGCTCGTAGTTGAATTT) was based on AMV4.5NF with the two end 3′ nucleotides removed. The TestPrime 1.0 program (https://www.arb-silva.de/search/testprime/) within the Silva database ([44], (> 3 K Mucoromycota reference sequences) was used to evaluate the coverage of Mucoromycota sub-phyla using the suggested settings of 1 mismatch with 5 bases 0-mismatch zone for a realistic simulation of PCR behaviour. This showed that the new primer AM-Sal-F improved coverage of both Glomeromycotina and Endogonales, whilst also allowing the inclusion of the Umbelopsidales group of the Mucoromycotina, and the Mortierellomycotina sub-phylum (Table S2).

Comparative analysis of the sequencing performance of AMV4.5NF and AM-Sal-F primers in combination with the Sato et al. [54] AMDGR reverse primer was performed (Fig. S3). Roots were collected from 3 independent locations separated by 10 m, within the 2 ha Boddington Meadow Nature Reserve, Northamptonshire (52° 10′ 24″ N, 1° 16′ 44″ W), UK in August 2019. Composite samples of root biomass (grasses and herbs) were collected from 0 to 10 cm depth at each location. The site has never been ploughed, is rich in wildflowers, is grazed periodically with sheep and cut for hay at the end of the growing season. DNA extraction, PCR amplification, sequence analysis and bioinformatic processing was performed as described above for the samples. Testing revealed that the AM-Sal-F primer enriched Endogonales sequences tenfold compared to AMV4.5NF, associated with a decrease in non-target sequences (e.g. Agaricomycetes), although this was associated with reduced % of Glomerales and an increase in the percentage of Mortierellales reads (Fig. S3).

A new primer set was devised to selectively amplify ~ 440 base pairs (bp) of the V4-6 region of the 18S rRNA gene of the Endogonales and related Mucoromycotina (further referred to as FRE-primers, Fig. S1). All Endogonales sequences (49) from the Silva database (July 2019) were aligned in MegAlign (DNASTAR Inc). Representative Glomeromycotina and other Mucoromycotina sequences were included. A forward primer was designed to a region downstream, but overlapping with the AM primer AMV4.5NF, which was distinct between the Glomeromycotina and the Mucoromycotina (6 nt mismatches within the 3′ region). The reverse primer had only 4 nt mismatches to the Glomeromycotina but in combination with the forward primer resulted in more specific amplification of the Endogonales/Mucoromycotina group. The FRE-F (GTTGAATTTTAGCCYTGGC) and FRE-R (CCCAAAAACTTTGATTTCTCW) primers amplify at 18S rRNA positions 619 and 1114 generating a 440 bp fragment.

Primer pairs were applied in separate PCRs using 15 ng of DNA in a master mix containing 12.5 µl 2 × Q5® High Fidelity Hot Start Master Mix (New England BioLabs® Inc., USA), 5 µl ddH_2_O and 1.25 µl of AM- (10 µM each) or FRE-primers (0.5 µM each) respectively. The PCR programme for both primer pairs started with denaturation at 98 °C for 30 s, followed by 35 cycles starting with polymerase activation at 98 °C for 10 s. Annealing of AM-primers was set at 60 °C for 15 s and for FRE-primers at 55 °C for 15 s. Both reactions were followed by elongation at 72 °C for 20 s. Final extension was conducted at 72 °C for 5 min. Size of amplicons was checked by gel electrophoresis. Following PCR, the DNA amplicons were purified using Agencourt AMPure XP beads (Beckman Coulter, Brea, CA, USA) according to the manufacturer’s instructions. The adapted amplicons were then modified by attaching indices and Illumina sequencing adapters using the Nextera XT Index Kit v.2 (Illumina, San Diego, CA, USA) by PCR as described in the manufacturer’s protocol. The amplicons were then purified and normalized using the SequalPrepTM Normalization Plate (96) Kit (Invitrogen) and quantitatively assessed using a Qubit 2.0 Fluorometer (Life Technologies). The final concentrations of the libraries was 4 nM. The libraries were sequenced using the MiSeq Reagent Kit v.3 600-cycle (Illumina) at The University of Warwick, UK.

Demultiplexed sequences with primer sequences removed were processed using the DADA2 pipeline [7] in Quantitative Insights into Microbial Ecology (QIIME 1.8., [8]). This pipeline removed low-quality reads (QC < 30), chimeras (using the consensus method) and singletons from the library. Taxonomy was assigned to amplicon sequencing variants (ASV) based on the SILVA132 2019 database [44]. ASV have been shown to outperform OTU based clustering methods when estimating the correct number of fungal species present in samples [22], and provide better reproducibility and finer granularity of compositional differences between samples, including improved detection of rare fungi [64]. Furthermore, ASV provide a better resource for comparisons across studies, which is particularly important for biogeographic analyses.

Sequencing of mycorrhizal communities in wheat roots with the AM primers (AM-Sal-F and AMDGR primers) which amplify both Mucoromycotina and Glomeromycotina yielded 300,371 sequences, and Mucoromycotina and Glomeromycotina accounted for 44% and 47.5% of all reads respectively. Only the sequences assigned to Mucoromycotina and Glomeromycotina were used for subsequent analyses. After removing reads from outside these groups, there were 241,089 sequences representing Mucoromycotina and Glomeromycotina, with between 9,381 and 33,529 reads per sample, assigned to 253 ASV (145 identified as Mucoromycotina and 108 as Glomeromycotina). BLAST using the nucleotide database Altschul et al. [6] was used to confirm correct taxonomic assignment of sequences. At the Order level, there were very few Diversisporales or Archaeosporales sequences, and no Paraglomerales sequences. All Mucoromycotina sequences were classified as Endogolales. There were approximately equal proportions of Endogonales and Glomerales sequences (Fig. S4).

The specific amplification of Mucoromycotina using FRE-primers produced 88,202 reads with 4230 to 11,895 reads per sample. After removing contaminants, the remaining 86,922 reads were grouped into 121 ASV which were solely assigned to the family Endogonaceae within the order Endogonales. Read numbers after filtering ranged from 3756 to 11,895 reads per sample. Only 5% of these sequences could be assigned to the genus *Endogone*, the remaining 95% were assigned to Endogonaceae (i.e. further taxonomic resolution was not possible).

## Statistical analysis

RStudio [36, 45] was used to calculate alpha diversity (species evenness, observed richness and Shannon’s diversity index) which were then compared among experimental treatments using Kruskal–Wallis tests. Reads for these analyses were rarefied to the lowest sample count (3756 and 9381 for the FRE and AM primers respectively). For analysis of beta diversity unrarified read counts were normalised using DESeq2 [31], and used to calculate a Bray Curtis dissimilarity matrix which was visualised using non-metric multidimensional scaling (NMDS) to identify groups of samples based on similar ASV compositions.

The effect of variety and N-addition on the ratios of Glomeromycotina to Mucoromycotina sequences was compared by analysis of variance (ANOVA) in linear mixed-effect models using the nlme-package [43]. For both datasets, the dispersion of homogeneity within treatment groups was assessed using the *betadisper*-function from the vegan-package [39]. Groups showing a sufficient homogeneity of variance (*p* ≥ 0.05) were run in permutational multivariate analysis of variance (PERMANOVA) using the *adonis*-function of the vegan-package. Blocks were included as random effects. Treatment groups showing significant differences at P < 0.05 were run through similarity percentage (SIMPER) analyses to identify ASV that contributed to these differences. The results of this method were validated by Kruskal–Wallis testing. All plots were generated with ggplot2 [70] in R, which was also used to correlate root colonisation data with ASV relative abundances of Glomeromycotina and Mucoromycotina in the samples.

### Phylogenetic analysis

The Mucoromycotina sequences amplified with the AM and FRE primers were aligned with a broad range of reference sequences, including environmental sequences which have been associated with FRE morphology in Australian samples. This included operational taxonomic units (OTUs) associated with FRE in *Trifolium subterraneum* pastures collected from across Australia [3] i.e. TS OTUs 7, 18, 43, 49, 110, 152, 289, 350, 432 and 980) and OTUs associated with FRE in *T. subterraneum* grown in pasture soil from Western Australia (i.e. KX434777, KX434773, KX434782, KX434776, KX434780 and KX434781) [40]. In particular, KX434777 is identical to the only described culture of an FRE forming fungus, *Planticonsortium tenue* [4]. Mucoromycotina sequences which were abundant in agricultural soils across Australia were also included [4], i.e. FREOZ OTUs 2, 43, 170. Additionally, we included sequences of Mucoromycotina associated with lower land plants (hornworts, and liverworts) (KC708405, KC708440, KC708398, KJ952232, KJ952217, KC708443, KC708419, KR779282, JF414224, JF414221, JF414209, LC429236). We included sequences from cultures of the saprotrophic fungi *Vinositunica radiata* (LC431088), *Sphaerocreas pubescens* (AB752291) and *Endogone pisiformis* (DQ322628) and a sporocarp of the saprophyte *Endogone lactiflua* (JF414204). A sequence of the putative ectomycorrhizal fungus *Endogone oregonensis* (JF414208) was also included. Glomeromycotan sequences including *Acaulospora laevis* (Y17633), Archaeospora (MH629023 and HF954887), *Diversispora aurantia* (AM713432), and a selection of ASV generated from our samples using AM primers (ASVs 451, 464, 415,270, 335, 323 and 254) were used as an outgroup.

Phylogenetic trees were built with MrBayes v.3.2.6 using a Bayesian phylogenetic inference approach [49] in which sequences of different lengths can be aligned by inserting gaps in the alignment to account for insertions or deletions in some sequences. This approach allowed us to align full 18S rRNA reference sequences together with the different sized AM and FRE sequences, and to identify sequences which were identical despite different sizes. Two separate MC3 runs with randomly generated starting trees were carried out for four million generations each with one cold and three heated chains. The evolutionary model applied a GTR substitution matrix, with a four-category autocorrelated gamma correction. All parameters were estimated from the data. The trees were sampled every 1000 generations and the first million generations discarded as burn-in. All phylogenetic analyses were carried out on the Cipres server [38]. The resulting phylogenetic tree was visualised in R using ggtree v3.6.2 [72], and posterior probability values > 0.8 are shown. The phylogenetic diversity accessed by the AM-primers and FRE-primers, independently, was estimated using Faith’s phylogenetic diversity metric.

## Results

### Mycorrhizal root colonisation

**Fig. 1:**
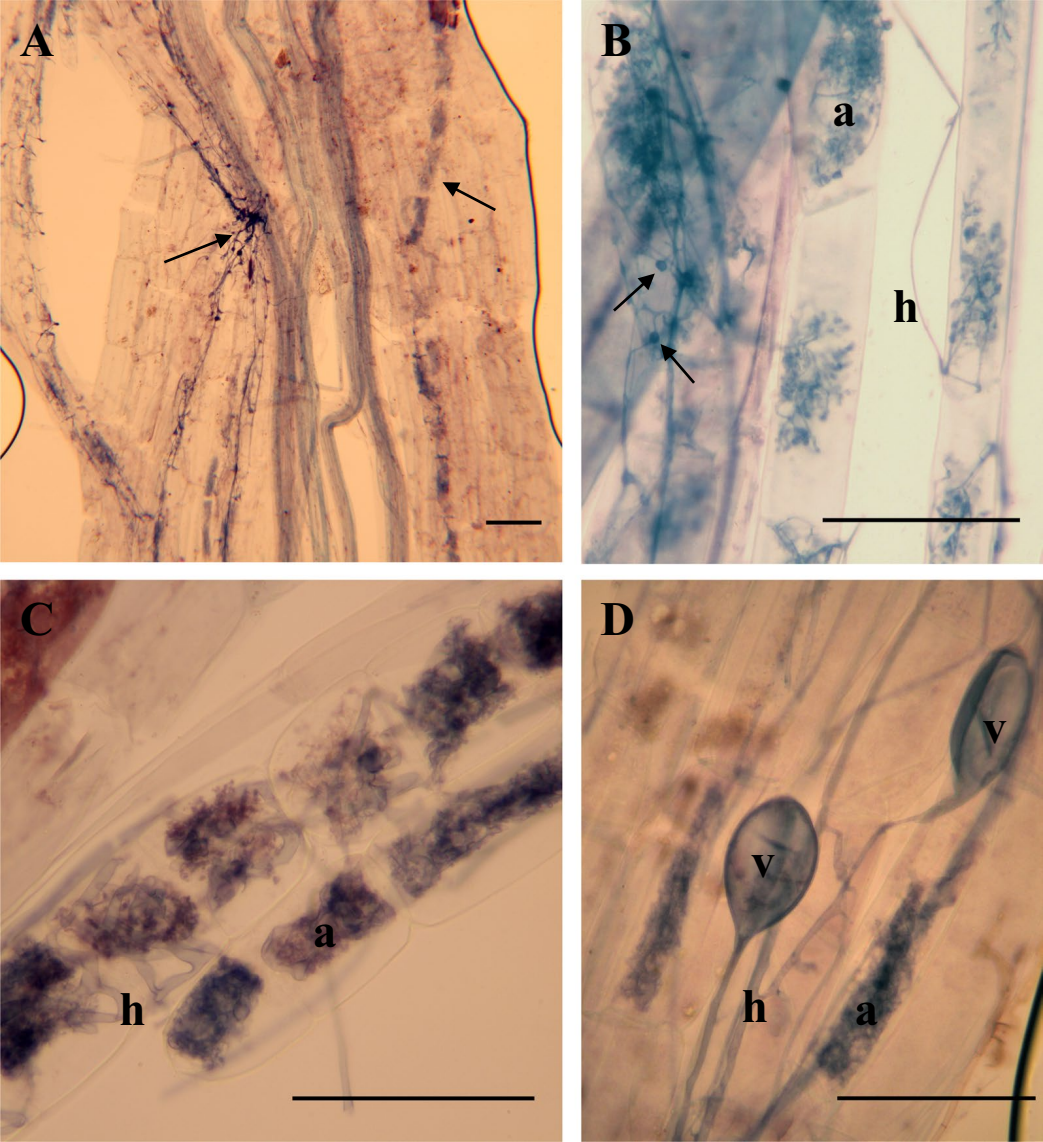
Microscopy images of fine root endophyte morphology linked to Mucormycotina arbuscular mycorrhizas (M-AMF) and coarse morphology associated with Glomeromycotina arbuscular mycorrhizas (G-AMF) in wheat roots. **A** Left arrow marks hyphae with intercalary swellings of G-AMF, right arrow marks arbuscules of G-AMF. **B** Arbuscules (a) and hyphae (h) of M-AMF, arrows point towards intercalary swellings. **C** Arbuscules and hyphae of G-AMF. **D** Vesicles (v), arbuscules and hyphae of G-AMF. Scale bars indicate 50 µm at ×10 (**A**) and ×40 (**B**–**D**) magnification

Root colonisation by AMF was high, with the frequency of colonisation (F%) around 60% and AM intensities (M%) between 33 and 37% (Table S3). There was no significant effect of N-addition or wheat variety on AMF colonisation frequency, intensity, or percentage of arbuscules, vesicles or hyphae. Wheat roots supported both coarse AMF and FRE morphologies indicating colonisation by both G-AMF and M-AMF (Fig. 1A–D). Visible FRE morphology included finely branched arbuscules and intercalary swellings on thin hyphae (Fig. 1A, B). Coarse and FRE colonisation was intermixed and it was not possible to produce an accurate assessment of the percentage root length colonised by each morphotype.

### Composition of Glomeromycotina and Mucoromycotina communities based on AM-primers

ANOVA showed that the relative abundances of Mucoromycotina and Glomeromycotina sequences (Table S4), and the ratio of Mucoromycotina to Glomeromycotina sequences (Table S5) were not significantly affected by variety or N-addition and there was no significant interaction between variety and N-addition. Alpha diversity of Mucoromycotina and Glomeromycotina was not significantly different between the varieties or N-treatments when analysed together (Fig. S5) or separately (Figs. S6, S7). Similarly PERMANOVA (Table S6) analysis showed that beta-diversity of the Glomeromycotina and Mucoromycotina communities when analysed together, or separately, was not significantly affected by either variety or N-addition. There was no significant correlation between AMF root colonisation and the relative abundance of Glomeromycotina (*R* = − 0.15, *p* = 0.63) or Mucoromycotina ASV (*R* = − 0.21, *p* = 0.5).

### Composition of Mucoromycotina communities using FRE-primers

**Fig. 2:**
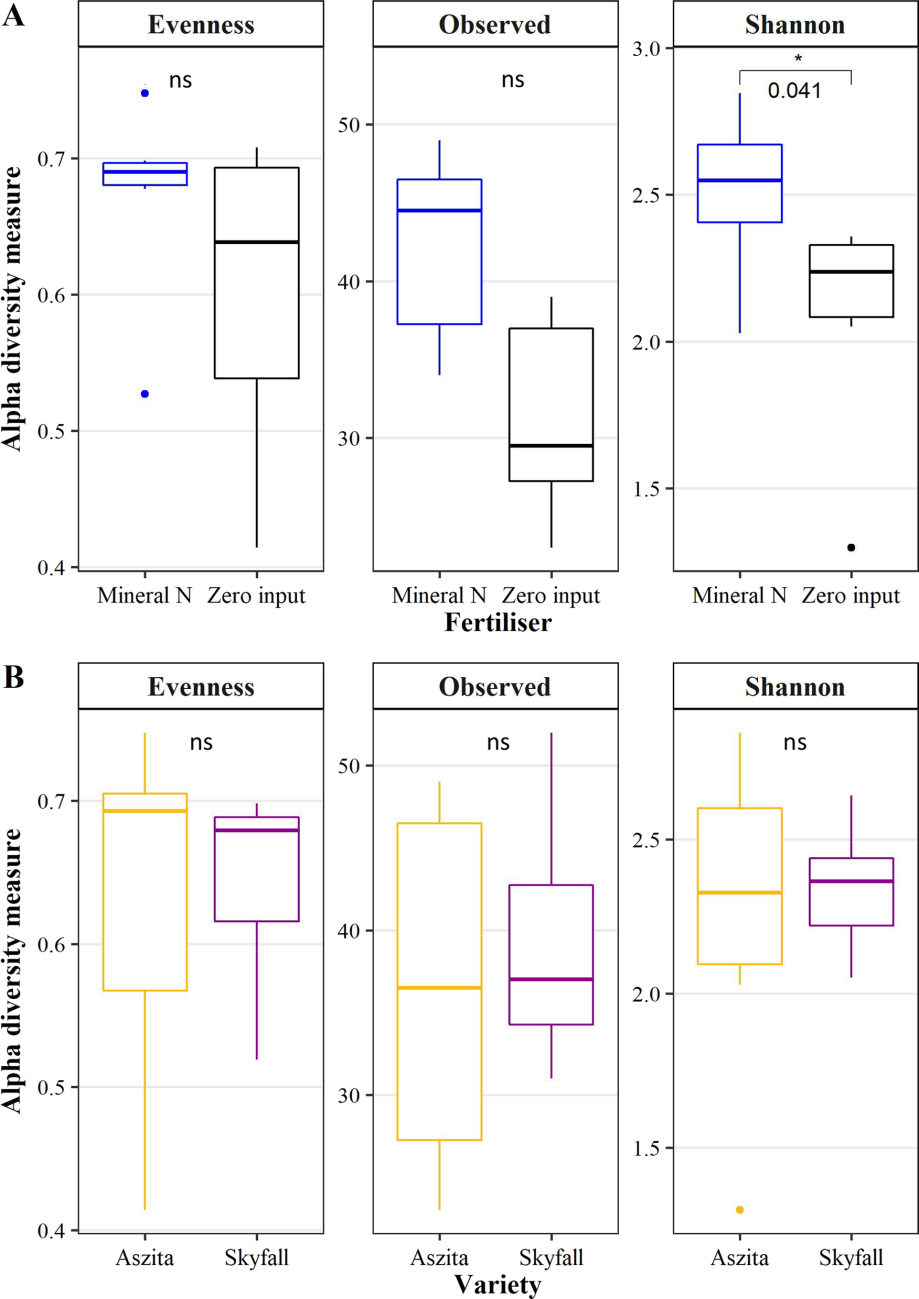
Box plot showing ASV evenness, number of observed ASV and Shannon diversity index of Mucoromycotina communities. Sequencing was performed using Mucoromycotina specific FRE primers. **A** Comparison of mineral N application with zero-input treatments (n = 6). **B** Comparison of wheat varieties (n = 6). Numbers indicate *p*-values for pairwise comparison by Kruskal–Wallis test (ns = not significant, * significant p ≤ 0.05)

**Fig. 3:**
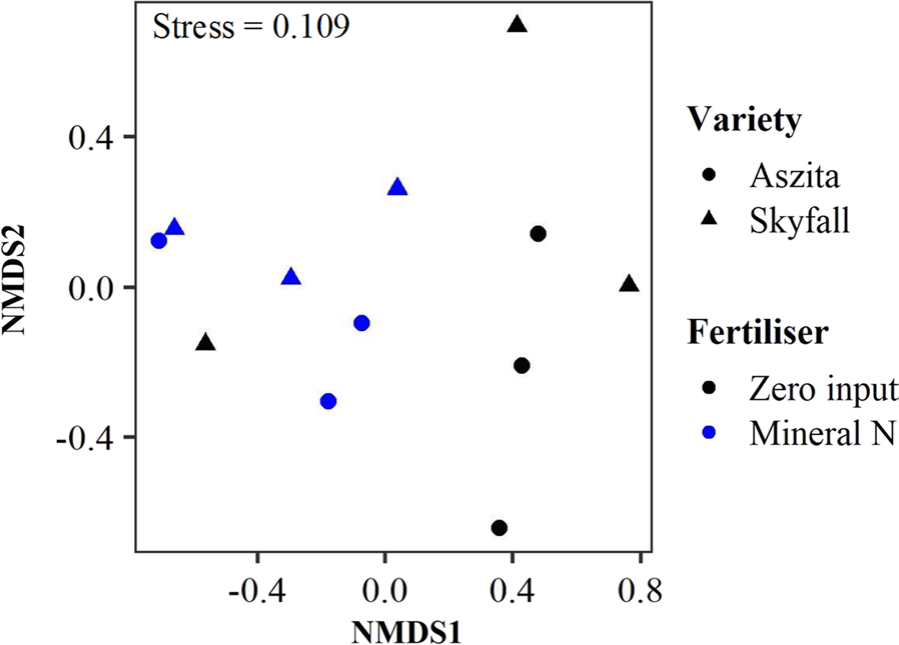
Non-metric multidimensional scaling plot of Bray Curtis dissimilarity of Mucoromycotina communities in roots of two wheat varieties with and without mineral-N application. Sequencing was performed using Mucoromycotina specific FRE primers

Shannon diversity of Mucoromycotina was significantly higher when mineral-N was applied, relative to the zero-N treatment (Fig. 2), and this was driven by trends in greater number of observed ASV, and greater eveness in the mineral-N relative to the zero-N treatment. However there was no significant difference in Shannon diversity between the two wheat hosts. PERMANOVA (Table 1) and NMDS (Fig. 3) analysis showed that beta-diversity of the Mucoromycotina community profile was significantly affected by N application, which contributed to 22% of community variation (P < 0.028). Wheat variety had no significant effect on beta diversity.

**Table 1:** Permutational multivariate analysis of variance of the effect of wheat variety (Aszita vs. Skyfall) and nitrogen treatment (mineral-N vs. zero-N treatments) on the composition of Mucoromycotina

	DF	SS	MS	F.Model	R^2^	Pr(> F)
Variety	1	778.5	778.47	0.68	0.055	0.719
Nitrogen	1	3137.7	3137.67	2.73	0.221	**0.028**
Variety:Nitrogen	1	1062.3	1062.32	0.92	0.075	0.406
Residuals	8	9209.8	1151.23	0.65		
Total	11	14,188.3	1			

Sequencing was performed using Mucoromycotina specific FRE primers. Bold indicates a significant effect

SIMPER analysis identified 12 ASV which contributed > 1% to the dissimilarity between Mucoromycotina communities in the zero-N and mineral N-addition treatments (Table 2), and for five of these ASV the relative abundance was also significantly different between the N treatments. ASV3 contributed 22.14% of the dissimilarity between N treatments. The relative abundance of this ASV was significantly (P < 0.05) increased by 20% in roots with mineral-N addition. ASV 7 contributed 4.63% of the variation in community composition between N treatments, with relative abundance increasing significantly (P < 0.05) from 1.51 in the zero-N treatment to 7.77% in the mineral-N treatment. ASV17, 18 and 21 contributed to less than 2% of the variation between N treatments, with significant (P < 0.05) increases in relative abundance from 0.38 to 0.53% in the zero-N treatment to between 1.75 and 2.56% in the mineral-N treatment. A number of further ASV contributed to the dissimilarity between N treatments (ASV1, 2, 8, 5, 10, 13, 15) but none of these showed a significant difference in relative abundance between treatments.

**Table 2:** Similarity percentage (SIMPER) analysis of ASV contributing to dissimilarity of Mucoromycotina communities in wheat roots from zero-N and mineral-N treatments (n = 6).

	% Relative abundance zero-N	% Relative abundance mineral-N	% Contribution to difference		P value
ASV3	9.39	29.99	22.14		0.034
ASV1	13.62	1.38	6.73		ns
ASV7	1.51	7.77	4.63		0.034
ASV2	18.71	11.07	4.17		ns
ASV8	10.60	2.81	3.60		ns
ASV5	13.34	10.58	3.28		ns
ASV13	5.68	0.24	1.89		ns
ASV15	0.82	2.76	1.81		ns
ASV17	0.48	2.56	1.59		0.022
ASV10	2.74	2.76	1.43		ns
ASV18	0.53	1.75	1.30		0.031
ASV21	0.38	2.26	1.20		0.010

P values were determined using Kruskal–Wallis tests

### Phylogenetic analysis of Mucoromycotina sequences

**Fig. 4:**
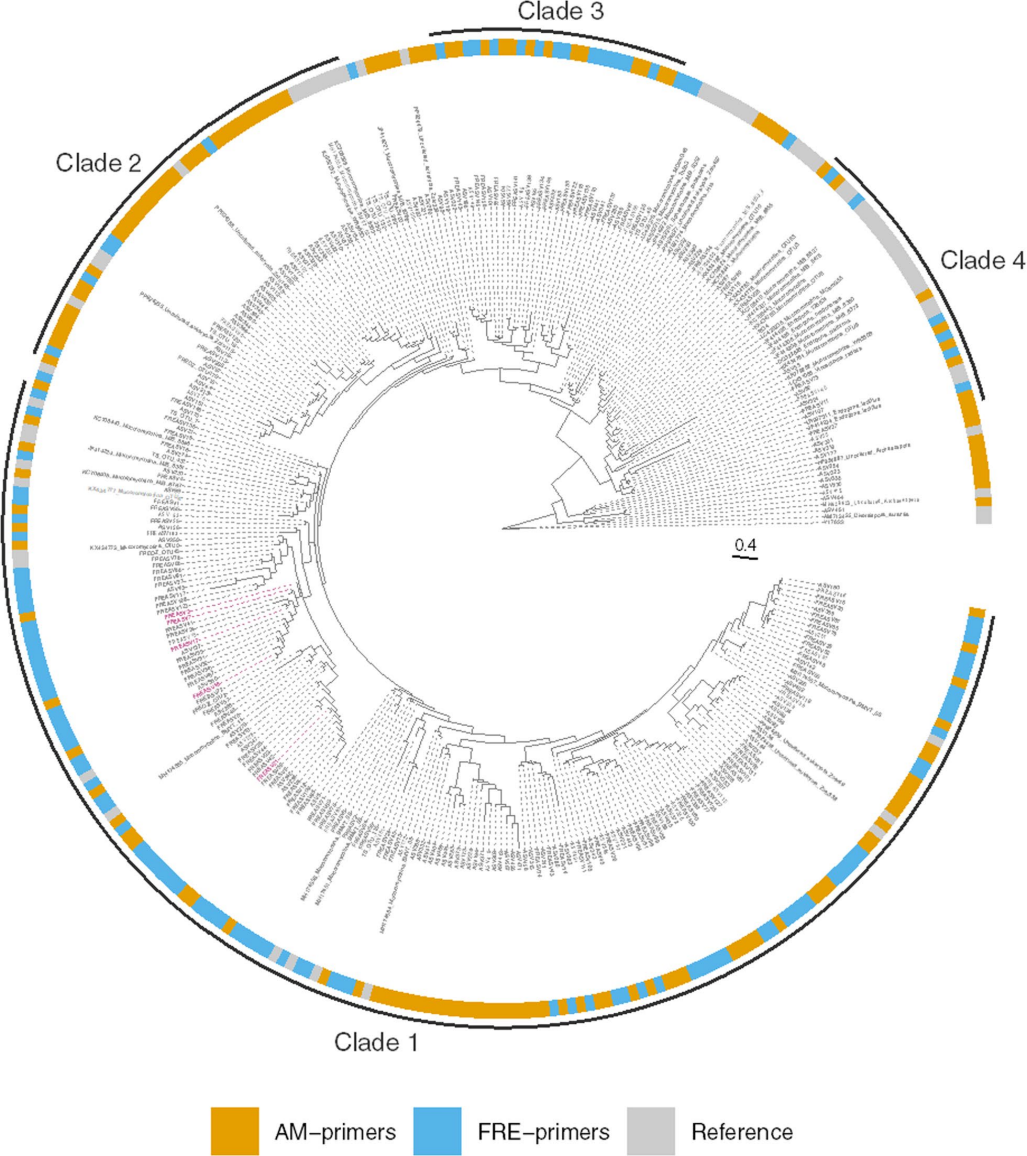
Phylogenetic assessment of ASV accessed using AM and FRE primers. Outer ring fill colour denotes sequence source. Clade labels denote the four main Mucoromycotina clades formed. The five FRE primer amplified ASV found to have significant differences in their relative abundance between mineral-N treatments are highlighted in pink. Branches with a grey circle have a posterior probability value > 0.8. Reference sequences are described in the Methods, section Phylogenetic analysis. Environmental sequences associated with fine root endophyte colonisation of roots include TS OTUs 7, 18, 49, 110, 152, 289, 350 and 432, from *Trifolium subterraneum* pastures across Australia [3] and sequences KX434777, KX434773, KX434782, KX434776, KX434780 and KX434781 from *T. subterraneum* grown in pasture soil from Western Australia [40]. Environmental sequence OTU4 from Orchard et al. [40], which shows 100% similarity to *Planticonsortium tenue* [4], is shown in bold (Clade 1)

A phylogenetic tree containing 145 ASV identified using AM primers, 121 ASV identified using FRE-primers, and reference Mucoromycotinian and Glomeromycotinian OTU sequences was visualised (Fig. 4). While this indicated that the two primer sets accessed a similar breadth of Mucoromycotinian diversity, Faith’s phylogenetic diversity was estimated to be greater for the ASV accessed using AM-primers (35.68) than those using FRE-primers (30.27). Nonetheless, the tree contained four main clades (referred to as Clades 1–4), with sub-clades largely represented by the ASV which were unique to each of the primer sets.

Clade 1 (Fig. 4, Fig. S8) included four of the five dominant sequences amplified with FRE primers (FREASV 1 (9.8% reads), 3 (22.3%), 5 (11.3%) and 8 (5.3%)) and three dominant ASV amplified by the AM-primers (ASV 17 (24.2%), 37 (9.1%) and 21 (6.0%)). This clade also contained sequence from the only described FRE culture, *Planticonsortium tenue* (KX434777), and other abundant sequences associated with FRE morphology from previous studies: KX434773 from glasshouse grown *T. subterraneum* [41], TS OTU43, OTU110 and TS OTU432 from pasture sites in Australia [3], and OTU2, from agricultural soils in Australia [4]. Further inspection of Clade 1 revealed the presence of three sub-clades: Clade 1a contained the five ASV which displayed significant differential abundance between mineral-N treatments. These sequences were found to have 97–98% similarity to one another.

Clade 2 included most of the reference sequences from Australian pastures, including TS OTU18, 152, 289, 350 and 980, and a sequence obtained from Australian agricultural soils (FREOZ OTU170). Clade 3 represented novel diversity. Lastly, Clade 4 represented the remaining low abundance sequences amplified with both primer sets, and was associated with ectomycorrhizal and saprotrophic Endogonales. Notably reference sequences obtained from hornworts and liverworts were also found in clades 1, 2 and 4.

## Discussion

In the current study we report findings using two new sets of primers. The AM-primer set is a modification of those published by Sato et al. [54], which co-amplify Mucoromycotina and Glomeromycotina, and have been used to profile putative M-AMF communities in previous studies [2–4, 33, 41]. The second, FRE-primers, specifically amplify Mucoromycotinian fungi, and provide a longer 440 bp sequence length than the Sato et al. [54] primers (220 bp). The AM-primers were used to investigate whether co-occurring G-AMF and M-AMF responded in a similar manner to different wheat varieties and N-addition, but neither factor had a significant effect on the diversity or composition of either Glomeromycotina or Mucoromycotina communities. Data generated with the FRE-primers also indicated no effect of wheat variety on Mucoromycotina community composition, but in contrast to the AM-primers, the FRE-primers indicated that N-addition induced changes to Mucoromycotina alpha and beta diversity, with marked effects on the relative abundance of a number of ASV. The dominant Mucoromycotina sequences amplified with both primer sets formed phylogenetic clades within the order Endogonales, along with sequences associated with FRE morphology from previous studies in Australia [3, 33, 41], and also the only described species of M-AMF to date, *Planticonsortium tenue*, isolated from New Zealand [4, 69].

The AM and FRE primer sets amplified similar richness of Mucoromycotina associated with wheat roots, although the AM-primers provided a greater phylogenetic range than the FRE-primers. Importantly, there were differences in the relative amplification of different phylogenetic clades between the primers. The FRE-primers detected shifts in community composition associated with N-addition that the AM-primers did not. This was shown to be the result of preferential amplification by the FRE-primers of a clade associated with FRE morphology in previous studies in Australia [3, 4, 33]. This highlights the importance of primer choice for characterising AMF communities and interpreting responses to environmental parameters. Similarly, a range of primer sets have been used to characterise G-AMF communities, and these have well known biases which can affect the evaluation of taxa richness and the relative abundance of families [23, 25].

Notably the AM-primers amplified a broad range of Mucoromycota in addition to Mucoromycotina, and furthermore this and earlier studies [3] show they provide very limited coverage of Diversisporales, Archaeosporales and Paraglomerales. Co-amplification of Mucoromycotina with other Mucoromycota sub phyla may be an advantage in some circumstances, such as to allow comparative understanding of ecological distribution patterns within the Mucoromycota. However, the limited coverage of Glomeromycota is problematic since Diversisporales, Archaeosporales and Paraglomerales are widely distributed and abundant, particularly within agricultural soils [16, 25]. Despite the longer fragment size amplified by the FRE primers, they provided less phylogenetic diversity than the AM primers, and in contrast to the AM primers, the FRE primers identified community responses to N application. To provide the most comprehensive analysis of AMF communities for metabarcoding analysis separate analyses of Glomeromycotina and Mucoromycotina is preferable. The use of widely used Glomeromycotina primers [25], together with our new Mucoromycotina specific FRE primers, provides the optimal approach.

The relatedness of Mucoromycotinian sequences detected in our study with the FRE forming *P. tenue* and Mucoromycotinian sequences associated with FRE morphology in Australia [3, 4, 33, 41], suggests a global distribution of Endogonales clades associated with FRE morphology. Despite being short amplicon sequences, several ASV were very close matches to sequences described from Australia, indicating that some M-AMF taxa could have a global distribution. Furthermore, several of the abundant Mucoromycotinian sequences we detected in roots showed close similarity to sequences associated with rhizoids of hornworts and liverworts, suggesting that these fungi could form both AM symbioses with higher plants and mycorrhiza-like associations with both early diverging vascular plants and non-vascular plants, akin to G-AMF. However, M-AMF sequences associated with non-vascular plants showed a broader phylogenetic distribution than the Mucormycotina sequences associated with FRE morphology from this study, and the earlier studies in Australia [3, 4, 33, 41].

In the current study, and in previous studies in which Mucoromycotina associated with FRE morphology have been characterised [3, 4, 33, 41], a diverse assemblage of Mucoromycotinian fungi has been detected. This could therefore suggest that there is a wide diversity of Mucoromycotina which can form AM associations, similar to G-AMF. Additionally, at some sites, such as the conventional high input agricultural location studied here, the richness of root associated Mucoromycotina ASV may even be higher than Glomeromycotina. To date only a single FRE species, *P. tenue*, has been described based on morphological evidence [69]. However, Thippayarugs et al. [62] identified a range of morphological characteristics which varied across published studies of FRE. They used 11 characteristics including hyphal surface smoothness, hyphal diameter and branching, vesicle diameter and shape, and staining intensity with trypan blue to differentiate five distinct FRE morphological groups colonising *Trifolium subterraneum* in Western Australia. There is a clear need to link molecular and morphological evidence to develop a taxonomic classification of M-AMF.

G-AMF may confer a range of benefits to their host, particularly P uptake, although they may also promote supply of other nutrients including N, calcium, magnesium and micronutrients. G-AMF may also provide the host with resistance against pests and disease, particularly soil-borne pathogens [15]. Understanding of functional diversity within G-AMF communities is limited, although there is evidence for trait-based variation between Glomeromycotina families. In particular, Gigasporaceae may form abundant extraradical mycelium which facilitates P uptake from soil and its subsequent translocation to the host. In contrast, Glomeraceae mycelium may mostly grow within the root, providing protection against soil-borne pathogens but lower benefits to host-P nutrition, while Acaulosporaceae may produce low hyphal biomass compared to the other groups, but equivalent P uptake to Glomeraceae, providing cost effective trade in C for P [32].

The functional significance of M-AMF is less clear, although there is evidence that M-AMF may provide N [20] and P to their host plant [41]. Comparative studies of Glomeromycotina and Mucoromycotina fungi associated with liverworts and hornworts showed that Mucoromycotina facilitated higher N uptake to the host, while the reverse was true for P. This could suggest complementary roles in nutrient acquisition by these groups [13]. The significance of diverse Mucoromycotina communities within roots, and their contribution towards the functional diversity of AM symbioses remains to be determined.

The tendency of G-AMF and M-AMF to intermingle in plant roots has frequently been described [21, 51, 71], and all samples analysed in the current study, using microscopy and molecular analysis, contained both AM groups. Albornoz et al. [3, 4] suggested that the two groups had overlapping ecological niches at the landscape scale, although there were different responses to temperature, pH and plant richness. In contrast to Glomeromycotina, nutrient availability was an important determinant of Mucoromycotina abundance, which increased with fertility.

In the current study, characterisation of Mucoromycotina with FRE-primers indicated shifts in community composition associated with N-addition, including changes to the dominant ASV. We have revealed that the response to N-addition was clearly linked to phylogeny, such that the five ASV with differential abundance between N treatments (ASV 3, 7, 17, 18 and 21) belonged to the same clade. Moreover, these ASV were closely related to one another within a sub-clade (Clade 1a) which was mostly accessed by the FRE-primers. The biological significance of these abundance shifts are unclear, however, since they could reflect direct impacts of N on Mucoromycotina, or indirect effects operating through the plant or other components of the root microbiome [42]. In contrast to G-AMF [67], agricultural management may favour M-AMF [4, 41, 56] and this could indicate a response to varied management practices including tillage and fertilisation.

Interestingly no effect of wheat variety was detected on either Glomeromycotina or Mucoromycotina communities. Previous studies indicated that Aszita and Skyfall supported high and low abundances of AM, respectively [11, 28, 63]. However, our data suggest that variety preferences for AM may vary across sites, determined by local climate, soil properties and management practices. This highlights the problems of extrapolating the outcomes of plant-AM interactions across locations, and for managing AM communities to support crop nutrition and system sustainability [50].

## Supplementary Information

Additional file 1.

Additional file 2.

## Data Availability

The raw sequence datasets and metadata reported in this study are available in the NCBI Sequence Read Archive under BioProject ID PRJNA1026851. The ASV sequences are deposited in the NCBI GenBank database under SUB13873553 (AM primers) and SUB13873261 (FRE primers).
