# Supplementary material for: New fungal primers reveal the diversity of Mucoromycotinian arbuscular mycorrhizal fungi and their response to nitrogen application
Source: Environ Microbiome. 2024 Sep 18;19:71. doi: 10.1186/s40793-024-00617-x (PMC11411812; doi:10.1186/s40793-024-00617-x)
Supplement: Supplementary file 1 — Additional file 1. [file 40793_2024_617_MOESM1_ESM.docx]

**Table S1** Agricultural management practices applied during the field trial.

| Application |  |
| --- | --- |
| **Previous crop** | Winter wheat |
| **Sowing date** | 26 September 2018 |
| **Seed rate** | Skyfall 400 seeds m^-2^ Aszita 550 seeds m^-2^ |
| **Crop emergence** | 15 November 2018 |
| **Herbicides** |  |
| **Autumn**  01/10/2018 | Liberator (400 g L^-1^ flufenacet and 100 g L^-1^ diflufenican) 0.6 L ha^-1^ |
| **Spring-Summer**  10/05/2019 | Axial (55 g L^-1^ pinoxaden) 0.3 L ha^-1^ Adigor (47 % w/w methylated rapeseed oil) 1 L ha^-1^, Fluroxypyr (200 g L^-1^, 20.4% w/w) 1 L ha^-1^ |
| **Fungicides** |  |
| **T1**  10/05/2019 | Turret (500 g L^-1^ (40.4% w/w) chlorothalonil) 2 L ha^-1^, Enterprise (140 g L^-1^ boscalid and 50 g L^-1^ epoxiconazole)  2.5 L ha^-1^ |
| **T2**  29/05/2019 | Ceratavo Plus (100 g L^-1^ benzovindiflupyr) 0.75 L ha^-1^, Daconil (500 g L^-1^ chlorothalonil) 2 L ha^-1^,  Epic (125 g L^-1^ epoxiconazole) 1 L ha^-1^ |
| **T3**  21/06/2019 | Kestrel (160 g L^-1^ (16.2% w/w) prothioconazole & 80 g L^-1^ (8.1% w/w) tebuconazole) 1 L ha^-1^ |
| **Plant growth regulators** applied at T1 | Chloromequat 1.25 L ha^-1^ |

**Table S2** Predicted coverage of AMV4.5NF and AM-Sal-F forward primers, in combination with the AMDGR reverse primer, across Mucoromycota groups. Data was generated with the Testprime 1 Program using the Silva 18S rRNA gene database.

|  | | | **% Coverage** | |
| --- | --- | --- | --- | --- |
| **Phylum** | **Sub-phylum** | **Order** | **AMV4.5NF** | **AM-Sal** |
| Mucoromycota |  |  | 46 | 64 |
| Mucoromycota | Glomeromycotina |  | 79 | 88 |
| Mucoromycota | Mortierellomycotina |  | 0 | 91 |
| Mucoromycota | Mucoromycotina |  | 0 | 17 |
| Mucoromycota | Mucoromycotina | Endogonales | 0 | 100 |
| Mucoromycota | Mucoromycotina | Mucorales | 0 | 0 |
| Mucoromycota | Mucoromycotina | Umbelopsidales | 0 | 100 |

|  | **F%** | **M%** | **A%** | **V%** | **H%** |
| --- | --- | --- | --- | --- | --- |
| **Variety** |  |  |  |  |  |
| Aszita(n = 6) | 63.3±7.1 | 33.8±7.47 | 19.2±3.41 | 2.5±2.47 | 21.2±4.79 |
| Skyfall (n = 6) | 62.8±6.41 | 37±5.57 | 23.4±4.88 | 3.7±1.2 | 21.1±2.2 |
| **Fertiliser** |  |  |  |  |  |
| Mineral N (n = 6) | 62.2±3.06 | 35.3±4.18 | 22.9±3.88 | 2.1±1.23 | 20.2±1.51 |
| Zero-input (n = 6) | 63.9±9.04 | 35.4±8.39 | 19.7±4.59 | 4±2.4 | 22.1±5.01 |

**Table S3** Root colonisation of Aszita and Skyfall wheat genotypes in zero-N and mineral-N treatments. Errors represent standard deviation. Colonisation was assessed according to Trouvelot et al. (1986). F, % frequency of mycorrhizal fragments; M, % mycorrhizal intensity; A, % Arbuscule, V, % Vesicle, H, % Hyphae.

**Table S4** % Relative abundance of Glomeromycotina and Mucoromycotina sequences in roots of Aszita and Skyfall wheat genotypes in zero-N and mineral-N treatments. Sequencing was performed using AM primers (AM-Sal-F forward and AMDGR reverse primers).

|  | **% Glomeromycotina** | **% Mucoromycotina** |
| --- | --- | --- |
| **Variety** |  |  |
| Aszita (n=6) | 43±6.2 | 47±3.8 |
| Skyfall (n=6) | 46±6.9 | 43±6.51 |
| **Nitrogen** |  |  |
| Mineral-N (n=6) | 45±7.5 | 45±4.3 |
| Zero-N (n=6) | 45±5.5 | 45±4.8 |

**Table S5.** Effect of variety and nitrogen treatment on the ratio of Glomeromycotina:Mucoromycotina sequences in wheat roots. Sequencing was performed with AM primers (AM-Sal-F forward and AMDGR reverse primers).

|  | Ratio |
| --- | --- |
| **Variety** |  |
| Aszita (n=6) | 1.01±0.194 |
| Skyfall (n=6) | 1.26±0.312 |
| **Nitrogen** |  |
| Mineral-N (n=6) | 1.17±0.304 |
| Zero-N (n=6) | 1.1±0.218 |
|  |  |

**Table S6.** Permutational multivariate analysis of variance of the effect of wheat variety and nitrogen treatment on the composition of combined Mucoromycotina and Glomeromycotina ASV, Mucoromycotina ASV only, and Glomeromycotina ASV only. Sequencing was performed with AM primers (AM-Sal forward and AMDGR reverse primers).

|  | **DF** | **SS** | **MS** | ***F*.Model** | **R^2^** | **Pr(>F)** |
| --- | --- | --- | --- | --- | --- | --- |
| **Combined** |  |  |  |  |  |  |
| Variety | 1 | 2910 | 2910 | 1.05 | 0.093 | 0.401 |
| Nitrogen | 1 | 3401 | 3401 | 1.23 | 0.109 | 0.120 |
| Variety:Nitrogen | 1 | 2934 | 2934 | 1.06 | 0.094 | 0.395 |
| Residuals | 8 | 22102 | 2763 | 0.71 |  |  |
| Total | 11 | 31346.9 | 1 |  |  |  |
| **Mucoromycotina** | |  |  |  |  |  |
| Variety | 1 | 1292 | 1292 | 1.00 | 0.091 | 0.595 |
| Nitrogen | 1 | 1554 | 1554 | 1.20 | 0.11 | 0.173 |
| Variety:Nitrogen | 1 | 1043 | 1043 | 0.81 | 0.073 | 0.815 |
| Residuals | 8 | 10330 | 1291 | 0.73 |  |  |
| Total | 11 | 14219 | 1 |  |  |  |
| **Glomermycotina** | |  |  |  |  |  |
| Variety | 1 | 223.2 | 223.2 | 0.93 | 0.085 | 0.713 |
| Nitrogen | 1 | 281.8 | 281.8 | 1.17 | 0.108 | 0.277 |
| Variety:Nitrogen | 1 | 188.2 | 188.2 | 0.78 | 0.072 | 0.848 |
| Residuals | 8 | 1920.6 | 240.1 | 0.74 |  |  |
| Total | 11 | 2613.82 | 1 |  |  |  |
