# Supplementary material for: New fungal primers reveal the diversity of Mucoromycotinian arbuscular mycorrhizal fungi and their response to nitrogen application
Source: Environ Microbiome. 2024 Sep 18;19:71. doi: 10.1186/s40793-024-00617-x (PMC11411812; doi:10.1186/s40793-024-00617-x)
Supplement: Supplementary file 2 — Additional file 2. [file 40793_2024_617_MOESM2_ESM.pptx]

## Slide 1
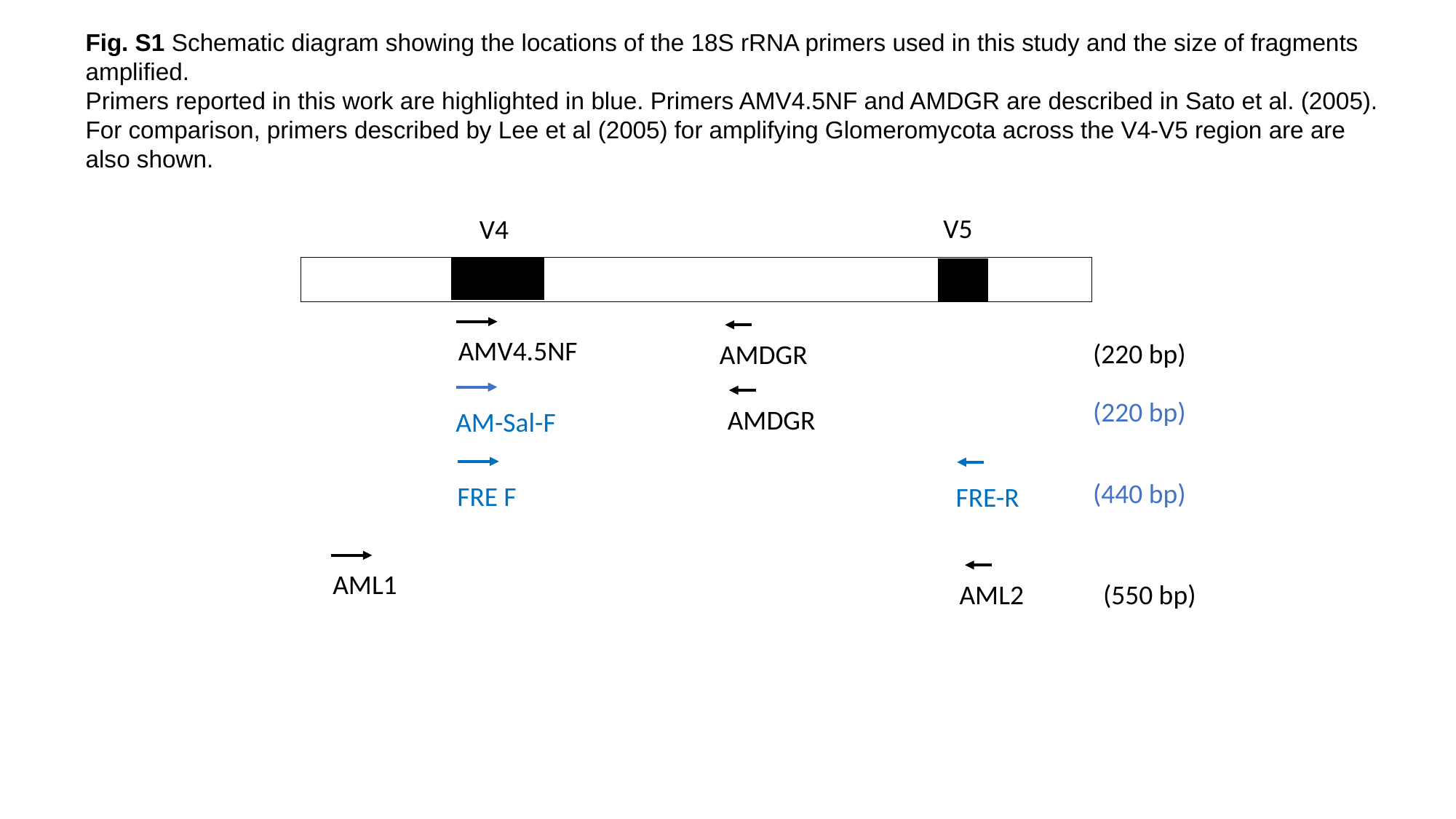

Fig. S1 Schematic diagram showing the locations of the 18S rRNA primers used in this study and the size of fragments amplified.
Primers reported in this work are highlighted in blue. Primers AMV4.5NF and AMDGR are described in Sato et al. (2005). For comparison, primers described by Lee et al (2005) for amplifying Glomeromycota across the V4-V5 region are are also shown.
V5
V4
AMV4.5NF
(220 bp)
AMDGR
(220 bp)
AMDGR
AM-Sal-F
(440 bp)
FRE F
FRE-R
AML1
AML2
(550 bp)

## Slide 2
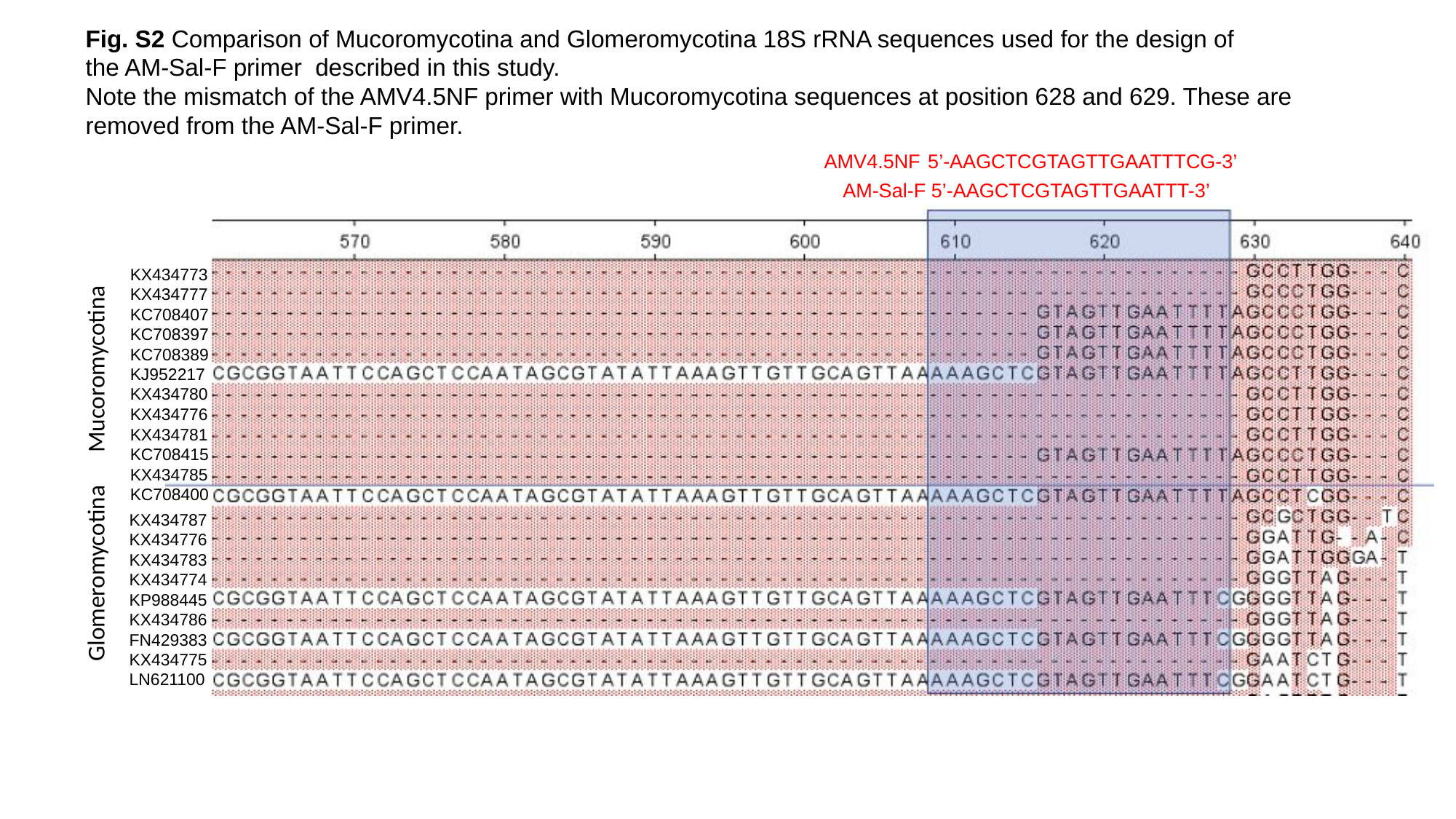

Fig. S2 Comparison of Mucoromycotina and Glomeromycotina 18S rRNA sequences used for the design of
the AM-Sal-F primer described in this study.
Note the mismatch of the AMV4.5NF primer with Mucoromycotina sequences at position 628 and 629. These are removed from the AM-Sal-F primer.
 AMV4.5NF	5’-AAGCTCGTAGTTGAATTTCG-3’
AM-Sal-F 5’-AAGCTCGTAGTTGAATTT-3’
KX434773
KX434777
KC708407
KC708397
KC708389
KJ952217
KX434780
KX434776
KX434781
KC708415
KX434785
KC708400
Glomeromycotina Mucoromycotina
KX434787
KX434776
KX434783
KX434774
KP988445
KX434786
FN429383
KX434775
LN621100

## Slide 3
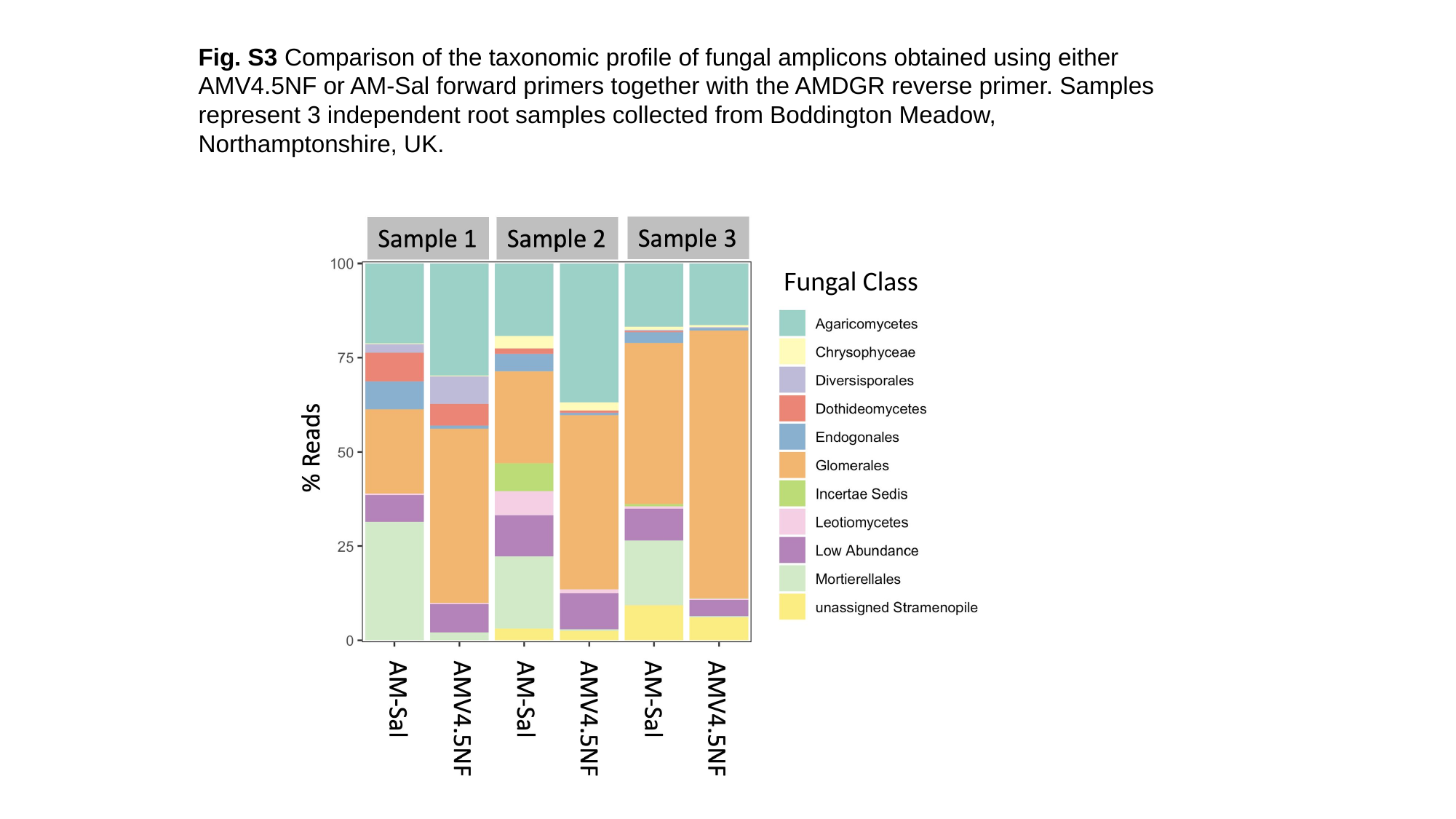

Fig. S3 Comparison of the taxonomic profile of fungal amplicons obtained using either AMV4.5NF or AM-Sal forward primers together with the AMDGR reverse primer. Samples represent 3 independent root samples collected from Boddington Meadow, Northamptonshire, UK.
Fungal Class

## Slide 4
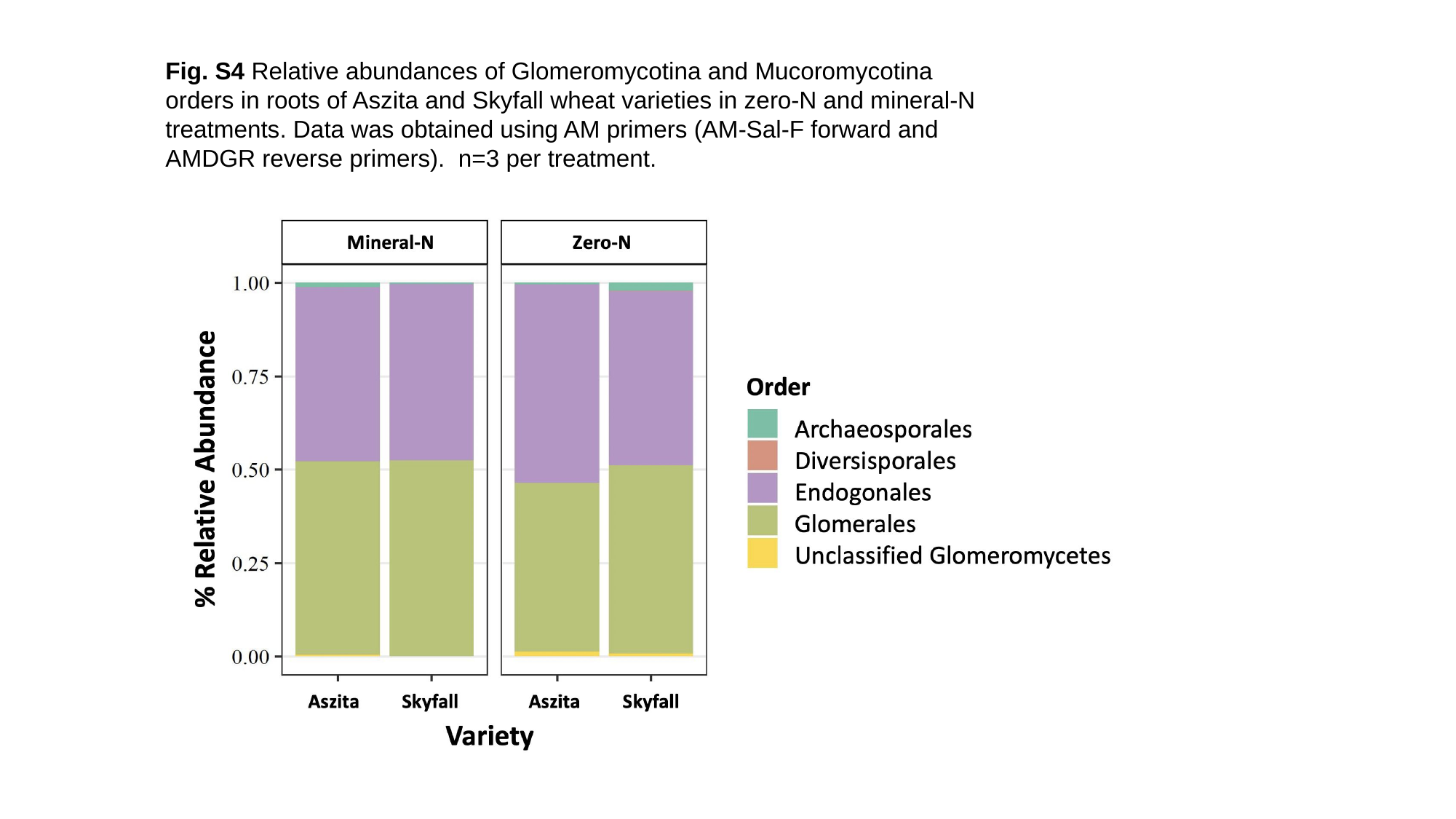

Fig. S4 Relative abundances of Glomeromycotina and Mucoromycotina orders in roots of Aszita and Skyfall wheat varieties in zero-N and mineral-N treatments. Data was obtained using AM primers (AM-Sal-F forward and AMDGR reverse primers). n=3 per treatment.

## Slide 5
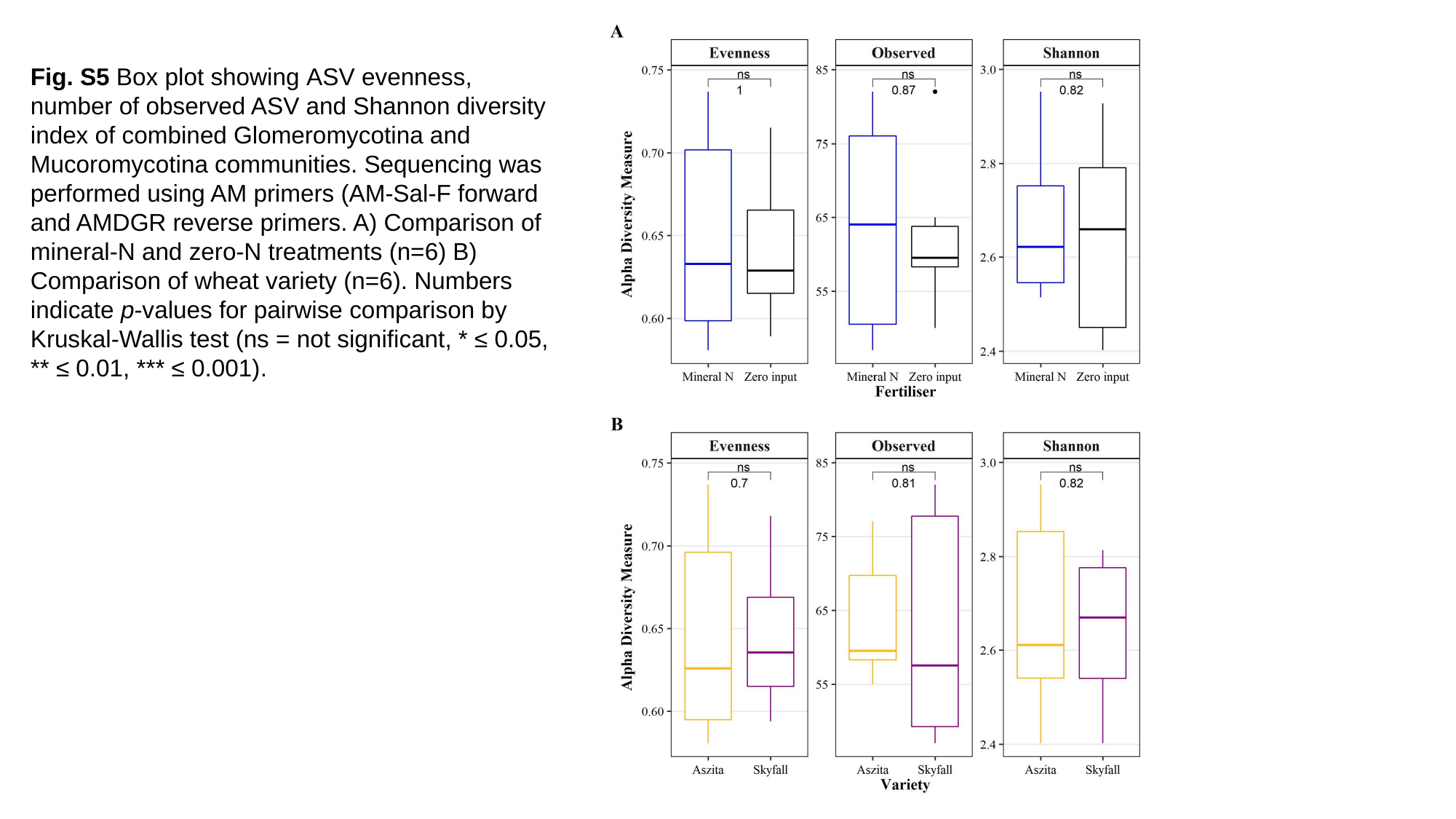

Fig. S5 Box plot showing ASV evenness, number of observed ASV and Shannon diversity index of combined Glomeromycotina and Mucoromycotina communities. Sequencing was performed using AM primers (AM-Sal-F forward and AMDGR reverse primers. A) Comparison of mineral-N and zero-N treatments (n=6) B) Comparison of wheat variety (n=6). Numbers indicate p-values for pairwise comparison by Kruskal-Wallis test (ns = not significant, * ≤ 0.05, ** ≤ 0.01, *** ≤ 0.001).

## Slide 6
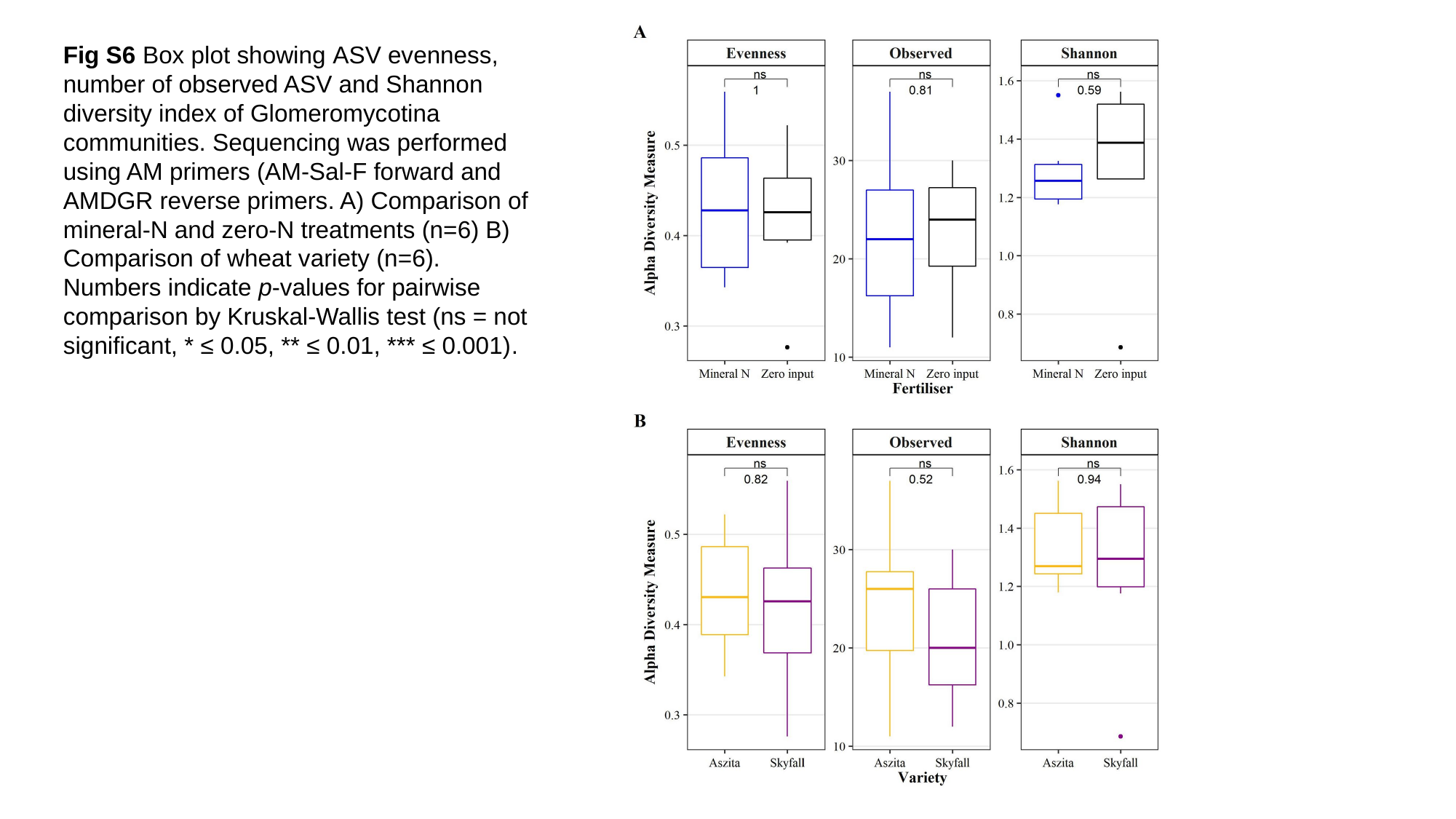

Fig S6 Box plot showing ASV evenness, number of observed ASV and Shannon diversity index of Glomeromycotina communities. Sequencing was performed using AM primers (AM-Sal-F forward and AMDGR reverse primers. A) Comparison of mineral-N and zero-N treatments (n=6) B) Comparison of wheat variety (n=6). Numbers indicate p-values for pairwise comparison by Kruskal-Wallis test (ns = not significant, * ≤ 0.05, ** ≤ 0.01, *** ≤ 0.001).

## Slide 7
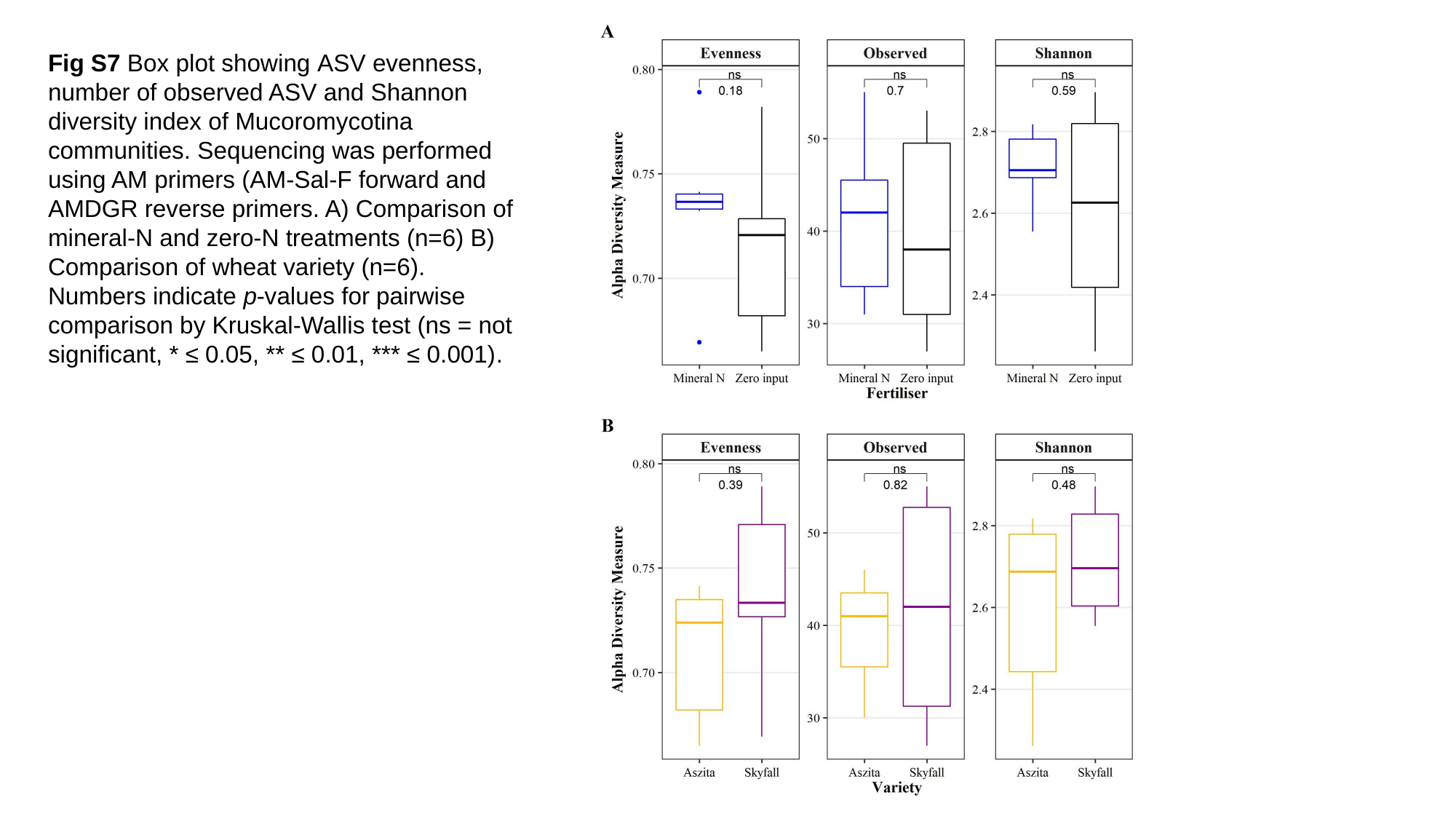

Fig S7 Box plot showing ASV evenness, number of observed ASV and Shannon diversity index of Mucoromycotina communities. Sequencing was performed using AM primers (AM-Sal-F forward and AMDGR reverse primers. A) Comparison of mineral-N and zero-N treatments (n=6) B) Comparison of wheat variety (n=6). Numbers indicate p-values for pairwise comparison by Kruskal-Wallis test (ns = not significant, * ≤ 0.05, ** ≤ 0.01, *** ≤ 0.001).

## Slide 8
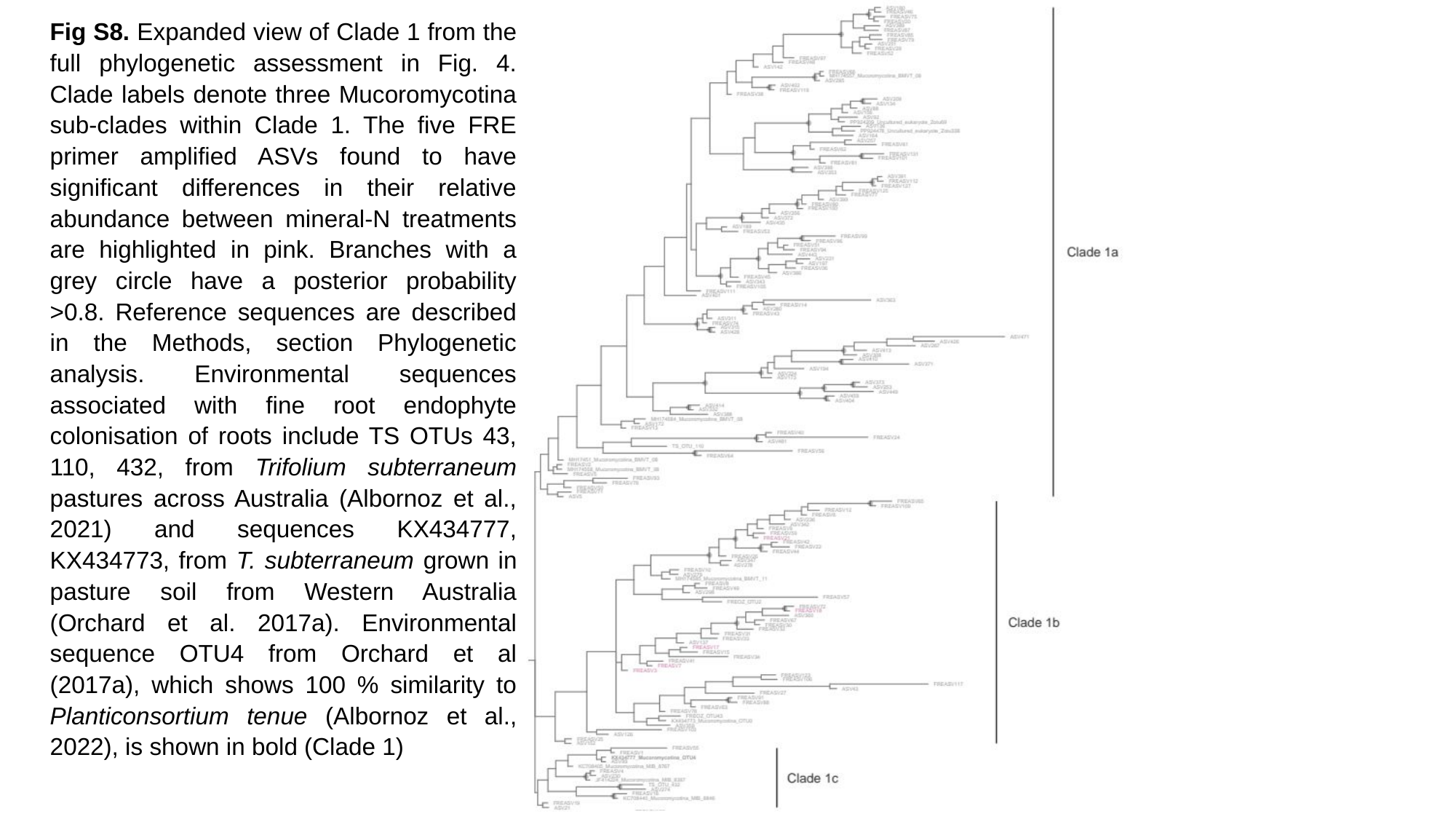

Fig S8. Expanded view of Clade 1 from the full phylogenetic assessment in Fig. 4. Clade labels denote three Mucoromycotina sub-clades within Clade 1. The five FRE primer amplified ASVs found to have significant differences in their relative abundance between mineral-N treatments are highlighted in pink. Branches with a grey circle have a posterior probability >0.8. Reference sequences are described in the Methods, section Phylogenetic analysis. Environmental sequences associated with fine root endophyte colonisation of roots include TS OTUs 43, 110, 432, from Trifolium subterraneum pastures across Australia (Albornoz et al., 2021) and sequences KX434777, KX434773, from T. subterraneum grown in pasture soil from Western Australia (Orchard et al. 2017a). Environmental sequence OTU4 from Orchard et al (2017a), which shows 100 % similarity to Planticonsortium tenue (Albornoz et al., 2022), is shown in bold (Clade 1)
